# Case Report: Surgical treatment of stage 3 medication-related osteonecrosis of the maxilla using the “FTT Split” surgical protocol: a report of 14 cases

**DOI:** 10.3389/froh.2025.1694766

**Published:** 2026-01-21

**Authors:** Daniel Jerković, Ante Mihovilović, Ante Pojatina, Tina Poklepović Peričić, Petra Stazić Kunčić

**Affiliations:** 1 Department of Maxillofacial Surgery, University Hospital of Split, School of Medicine, University of Split, Split, Croatia; 2 Department of Maxillofacial Surgery, University Hospital of Split, Split, Croatia; 3 Department of Prosthodontics, Study of Dental Medicine, University of Split School Medicine, Split, Croatia

**Keywords:** bisphosphonates, maxillectomy, medication-related osteonecrosis of the jaw, oral surgery, oroantral communication, surgical therapy

## Abstract

Most severe presentation of medication-related osteonecrosis of the jaw (MRONJ) is classified as stage 3 for which there are still no standard surgical therapy guideline. Our aim was to describe “Five Times Three (FTT) Split” protocol as surgical treatment of stage 3 maxillary MRONJ. The present case series included a total of 14 patients diagnosed with stage 3 MRONJ of the upper jaw who were treated from February 2022 to September 2024 with mucoperiosteal flap and “FTT Split” protocol. The patients were followed up both clinically and radiologically for a period ranging from 9 months to 3 years. Primary wound closure was successfully achieved in all cases without any signs of oroantral or oronasal communication, and complete healing was observed during the postoperative follow-up period. The “FTT Split” surgical protocol demonstrated high success rates during a controlled postoperative period.

## 1 Introduction

Medication-related osteonecrosis of the jaw (MRONJ) is a pathological condition that affects either the upper or lower jaw and is associated with the use of antiresorptive or antiangiogenic agents. It is a serious condition that significantly reduces the quality of life, characterized by the non-healing exposure of the jawbone for at least 8 weeks in patients with a history of prior use of antiresorptive or antiangiogenic drugs, in the absence of head and neck radiation exposure (1–3). Maxilla is less frequently affected with MRONJ than the mandible, as the maxillary cancellous bone has better vascularization compared to the dense, compact bone of the mandible (4). Treatment of MRONJ is typically guided by the American Association of Oral and Maxillofacial Surgeons (AAOMS) guidelines, which outline specific therapeutic approaches for each stage of the disease (5). Since there is higher incidence of MRONJ in the mandible, most published case series primarily focus on surgical treatments of the lower jaw. However, treating the maxilla is more challenging due to its proximity to the maxillary sinus and the potential for rapid progression (4). Stage 3 clinical presentation includes exposed or necrotic bone, or fistulae probing to bone with signs of infection, along with one or more of the following: necrosis extending beyond the alveolar bone (e.g., into the maxillary sinus or zygoma), pathological fracture, extraoral fistula, oroantral/oronasal communication, or osteolysis reaching the inferior border of the mandible or the sinus floor (5).

The key objective of the surgery is to halt the progression of the disease and ensure primary healing of the mucosa, while preventing any communication between the oral cavity and nasal cavity or maxillary sinus (5). Literature suggests that a mucoperiosteal flap is not the preferred method for treating large MRONJ lesions of the maxilla, particularly when there is involvement of the maxillary sinus (4, 6). For this reason, we aim to present our experience in treating stage 3 maxillary MRONJ using a mucoperiosteal flap with “Five Times Three (FTT) Split” protocol.

## 2 Patients and methods

The present case series included 14 patients with stage 3 MRONJ of the maxilla who were treated from February 2022 to September 2024 at the Department of Maxillofacial Surgery, University Hospital of Split. All 14 patients had exposure of necrotic bone affecting maxillary alveolar process, accompanied by pain and infection, presence of extensive oroantral or oronasal communication, along with sinus floor osteolysis and inflammation of the maxillary sinus mucosa. All of them were on intravenous bisphosphonate therapy and were indicated for partial/total maxillectomy. None of the patients received any therapy prior our surgical treatment. All fourteen patients provided written informed consent.

### 2.1 Surgical procedure

**Figure 1:**
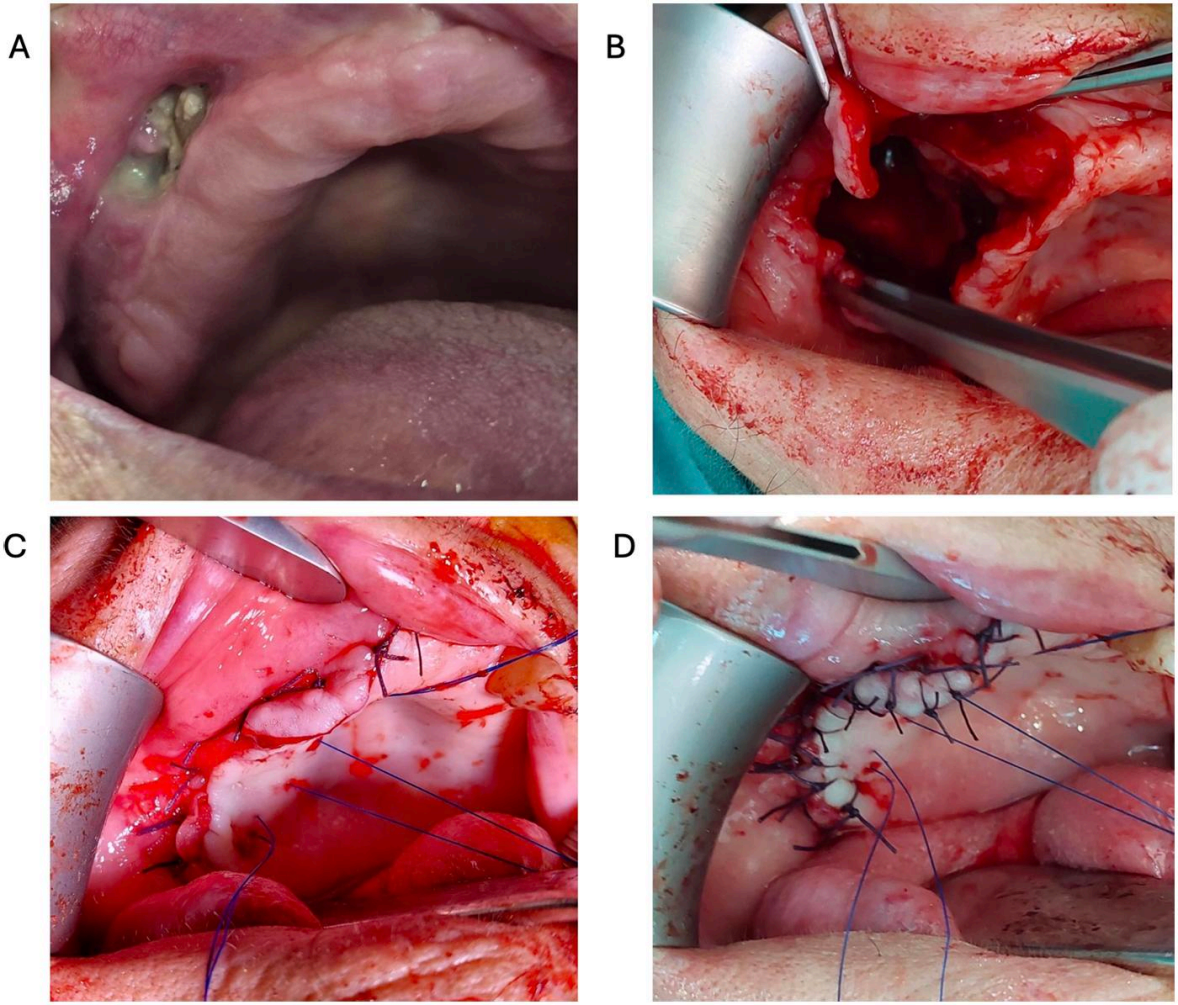
Surgical procedure. **(A)** The exposed necrotic bone, **(B)** Defect after removal of bone sequesters and necrotic bone, **(C)** Vertical mattress absorbable sutures ensuring eversion of the edges and three non-absorbable palatal sutures for tension reduction, **(D)** Individual single absorbable sutures.

To establish the diagnosis, medical history analysis, clinical and radiological examination [orthopantomography and cone beam/multi slice computed tomography (CBCT/MSCT)], and a preoperative biopsy were performed. In ten patients, bisphosphonate therapy was discontinued prior to surgery following multidisciplinary consultations with hematology and oncology specialists. In the remaining four cases, continuation of bisphosphonate treatment was deemed necessary due to the underlying malignancy. Microbiological smears were collected preoperatively and peroral antibiotics were prescribed based on sensitivity testing for 3–5 days prior to surgery. Good sensitivity to amoxicillin/clavulanic acid (875 mg/125 mg) was observed in all 14 patients and intravenous antibiotic therapy was initiated accordingly one day prior to surgery and continued for up to 5 days postoperatively. Additionally, all patients were instructed to rinse their mouths with 0.12% chlorhexidine (Miradent, Mouth Rinse Paragard CHX, Hager Pharma GmbH, Duisburg, Germany) two to three times daily for one minute, starting 10 days prior to surgery and continuing for two weeks postoperatively. All patients underwent surgery under general anesthesia and surgical therapy was performed in the same manner in all patients. Following the induction of anesthesia, anemization was performed using 2 mL local anesthetic suspension containing 40 mg of articaine hydrochloride and 0.005 mg of adrenalin in the form of adrenaline hydrochloride (Ubistesin, 3M ESPE, Neuss, Germany). The surgical field was washed with antiseptic solution containing 1 mg/mL octenidine dichloride + 20 mg/mL phenoxyethanol (Octenisept, Schülke & Mayr GmbH, Norderstedt, Germany). Prior to elevating the Nowak-Peter flap, the mucosa surrounding the exposed bone was excised (Figure 1A). Once the Nowak Peter flap was elevated, bone sequesters and necrotic bone were removed to macroscopically visible healthy bone (Figure 1B). The inflammatory mucosa of the maxillary sinus was removed and the maxillary sinus was artificially opened through the inferior nasal passage (Caldwell-Luc) (7). The Nowak-Peter flap was then converted into a Wasmund flap by incising the periosteum high in the vestibular fornix. The mucosa of the hard palate was split to a maximum thickness of 3 mm to allow eversion of the edges of the vestibular and palatal mucosa during suturing. The maxillary sinus was packed with iodoform gauze, which was pulled through the artificially created inferior nasal passage. After ensuring the mobility of the vestibular and palatal mucosa, a meticulous suturing procedure was performed. This approach aimed to achieve a tension-free, watertight closure of the wound, effectively sealing the extensive communication between the oral and nasal/sinus cavity.

First, three non-absorbable polypropylene palatal sutures, size 3-0 and 4-0 (ETHICON, Johnson & Johnson Medical Ltd. Simpson Parkway, Krikton Campus, Livingston, UK) were placed to reduce tension and were temporarily secured with hemostatic forceps. After the palatal sutures were in place, vertical mattress absorbable 3-0 sutures, polyglactin 910 (Vycril ETHICON, Johnson & Johnson Medical Ltd. Simpson Parkway, Krikton Campus, Livingston, UK) were used to close the mucosal edges, ensuring eversion of the edges (Figure 1C). Once the edges were properly everted, individual single absorbable 4-0 sutures, polyglactin 910, were applied (Vycryl ETHICON, Johnson & Johnson Medical Ltd. Simpson Parkway, Krikton Campus, Livingston, UK) (Figure 1D). After positioning the flap, the previously placed palatal sutures were tightened and secured. To promote optimal healing, a nasogastric tube was inserted.

**Figure 2:**
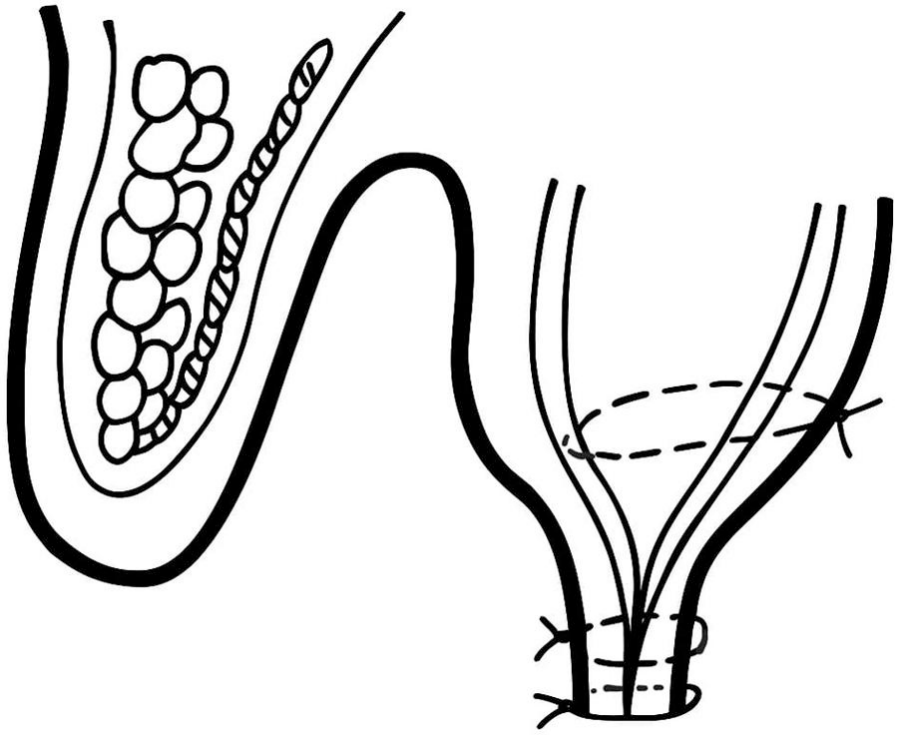
Schematic view of sutures in “FTT Split” (five times three split) protocol.

We refer to the surgical technique as “FTT Split” (Five Times Three Split) protocol (Figure 2), which consists of the following components:

Three rows of sutures.
Three palatal periosteal sutures for half of the palate.
A split palatal mucosa with a maximum thickness of three millimeters.
A suture removal period of three weeks.
A three-millimeter contact between the everted palatal and buccal mucosa.

This structured approach ensures both clarity and precision throughout the procedure. The color, texture, temperature, and degree of swelling of the flap were closely monitored. A liquid diet was provided through the nasogastric tube. All patients postoperatively received antibiotic, corticosteroid, and gastroprotective therapy. The nasogastric tube was removed 7 days post-surgery, absorbable sutures were removed 14 days postoperatively, and the palatal sutures were removed three weeks after the procedure. After the operation, the pathologist re-examined 14 bone biopsies. All findings were consistent with the diagnosis of MRONJ, which ruled out malignancy.

### 2.2 Follow-up

**Figure 3:**
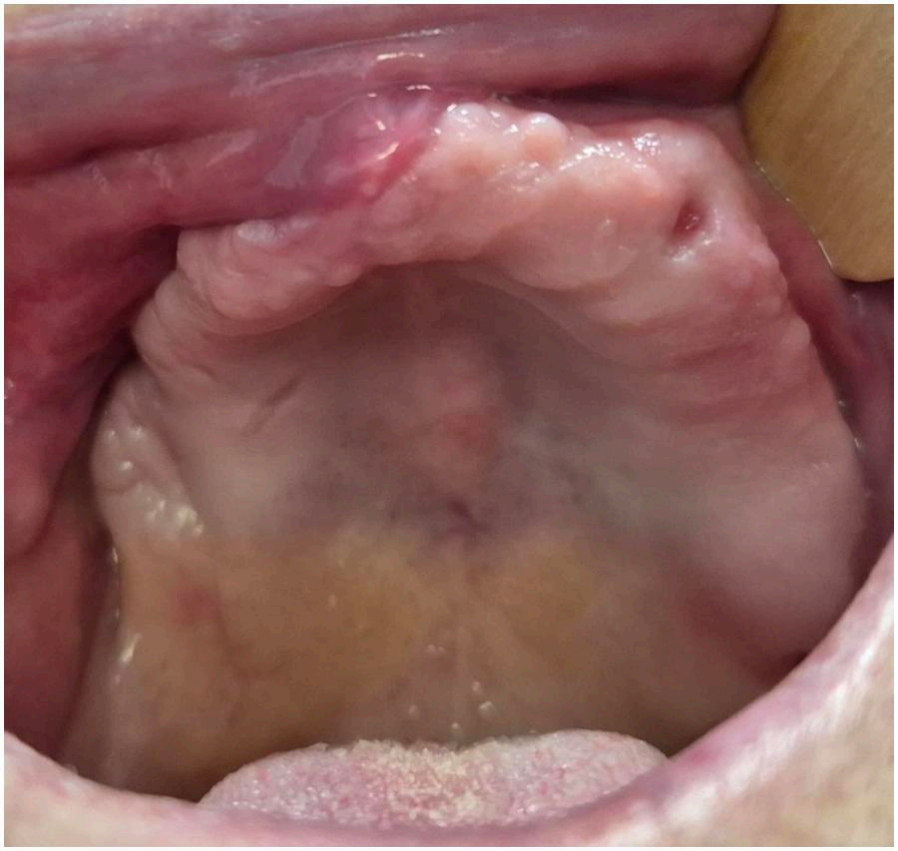
Postoperative healing after 9 months.

**Figure 4:**
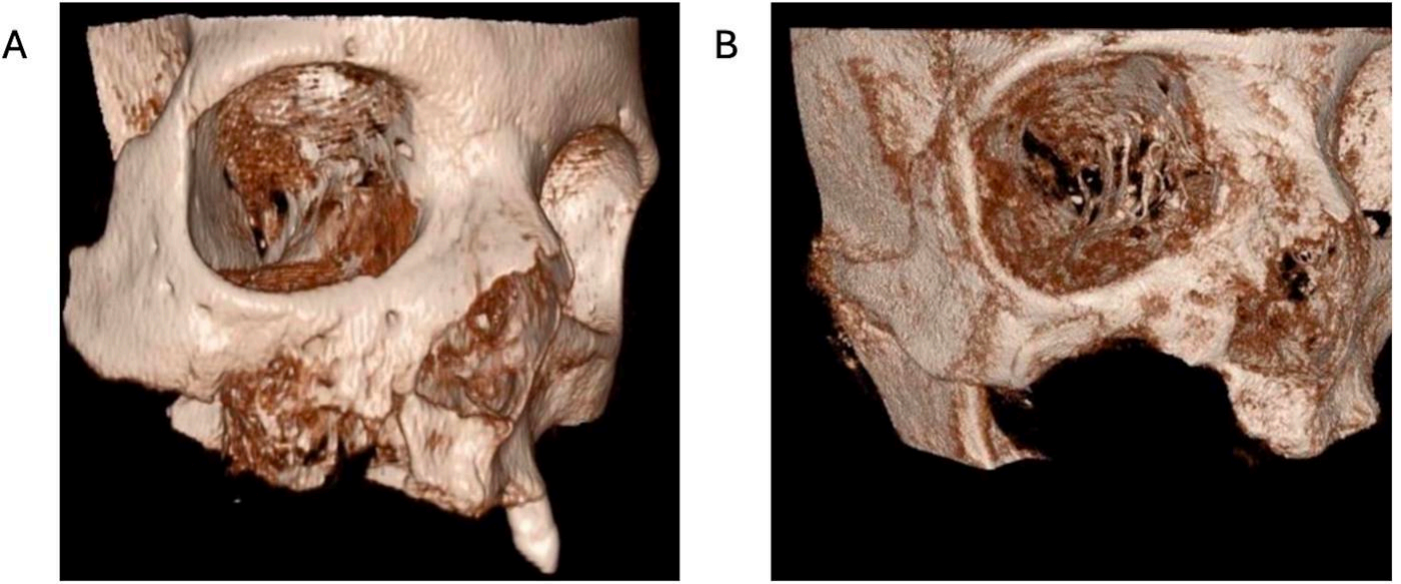
CBCT image. **(A)** Before surgical treatment **(B)** After 9 months.

This case series included 14 patients (10 females and 4 males), aged between 55 and 71 years (mean age 63.0 ± 5.55 years), who underwent surgery for the treatment of maxillary MRONJ under general anesthesia using the “FTT Split” technique (Table 1). Bisphosphonates were used in three patients with bone metastases from breast and prostate cancer, and in the others for the treatment of multiple myeloma and lymphoma. The most used agents were zoledronic acid (zoledronate) at a dose of 4 mg intravenously every 4 weeks and pamidronate at a dose of 90 mg intravenously every 4 weeks, for a duration of 16–48 months, depending on the underlying disease and therapeutic response. In all cases, bisphosphonate therapy was administered in combination with corticosteroids. In addition to the expected postoperative complications, including swelling and pain, eight patients experienced postoperative anesthesia/paresthesia in the area innervated by the infraorbital nerve. Of these eight patients, five reported complete resolution of symptoms within six months. Two patients reported significant reduction in the intensity of anesthesia/paresthesia during the follow-up period, while one patient showed no change in symptoms. Treatment was considered successful if during the follow-up period there was no disease recurrence and development of oroantral/oronasal communication. The follow-up period was from 10 months to 3 years and included control radiographic images (Figures 3, 4). All patients were provided with removable prosthetic replacements which enabled adequate masticatory, phonetic and esthetic function. All patients were satisfied with the outcomes of surgical therapy and stated that their quality of life was significantly improved.

**Table 1:** Patients’ characteristics and duration of follow up.

Patient	Age	Gender	Medical history	Type of bisphosphonate	Duration of bisphosphonate therapy (months)	Follow up (months)
1	55	Female	Lymphoma	Zoledronate	40	10
2	71	Female	Multiple myeloma	Pamidronate	22	16
3	56	Male	Prostate cancer	Zoledronate	20	22
4	70	Female	Multiple myeloma	Zoledronate	29	36
5	57	Male	Prostate cancer	Zoledronate	32	30
6	69	Female	Multiple myeloma	Pamidronate and Zoledronate	48	20
7	58	Female	Lymphoma	Zoledronate	32	30
8	68	Male	Lymphoma	Zoledronate	21	34
9	59	Female	Breast cancer	Zoledronate	36	28
10	67	Female	Multiple myeloma	Pamidronate	27	32
11	60	Female	Lymphoma	Zoledronate	32	24
12	66	Male	Lymphoma	Zoledronate	16	28
13	62	Female	Lymphoma	Zoledronate	18	26
14	64	Female	Multiple myeloma	Pamidronate	24	10
			Patients (*N* = 14)			
Age			Median 63, range 55–71			
Duration of bisphosphonate therapy (months)			Median 28, range 16–48			
Duration of follow up (months)			Median 27, range 10–36			

## 3 Discussion

Medication-related osteonecrosis of the jaw remains a disease of unknown etiology (8, 9). As such, the optimal treatment approach remains uncertain, and there is no consensus on the best choice for treatment. The primary goal of treating MRONJ is to halt the progression of the disease. According to some authors, surgical treatment should be the first choice since it offers better predictability and higher success rates, even in advanced stages (6, 10). According to Giudice et al. (11) early surgical intervention can help prevent the silent progression of the disease by reducing the duration for which antiresorptive treatment is withheld. Moreover, surgical treatment should be considered even in the early stages (stages 1 and 2) to prevent further lesion progression and complications. Besides, a precise histopathological analysis of the enucleated sequesters and resected necrotic bone can be performed after surgery, which helps rule out possible metastases and other malignancies (4, 8, 10–13).

While the maxilla is less frequently affected by MRONJ, it presents significant clinical challenges. These include its extension into the maxillary sinus, potential communication with the oral cavity, and possible complications such as nasal septal abscess, orbital cellulitis, skull base necrosis, and brain abscess (14–17). Therefore, clinicians must be familiar with the early clinical and radiographic manifestations of maxillary MRONJ disease in order to diagnose and treat the disease promptly (18).

Maxillary sinus involvement was reported in 35.8% of maxillary lesions (18), while Aljohani et al. (4) and Wasserzug et al. (19) reported the presence of an oroantral fistula in 32% and 38% of cases, respectively. Closing oroantral (OA) and oronasal communications is a crucial surgical procedure, and its success has a significant impact on the patient's functionality and quality of life. Maxillary defects and oronasal/oroantral communications can be reconstructed using regional or distant flaps (e.g., mucoperiosteal flaps, free flaps, buccal fat pad flap, nasolabial flap) or rehabilitated with a prosthetic obturator. Regional flaps are more commonly used than free flaps for closing oroantral communications due to the higher complication rates associated with free flaps, which, although effective, increase comorbidity, especially in elderly and terminal patients. While obturators provide satisfactory aesthetics and function, and help prevent nasal fluid leakage, their inadequate retention and stabilization can increase the risk of MRONJ recurrence due to trauma caused by the prosthesis (4, 20, 21).

The use of mucoperiosteal flap is not well-established or widely accepted in the management of MRONJ stage 3 and for the closure of extensive oroantral/oronasal communication. It is a conventional, convenient, and effective technique typically used for managing MRONJ lesions at stages 1 and 2, as it is considered most suitable when the surrounding mucosa is relatively healthy and there is no communication with the maxillary sinus (4, 6). However, at stage 3 of MRONJ, as presented in our case series, a wide oroantral communication often occurs following the extensive removal of necrotic bone and sequestra. This makes the primary closure of the defect and communication more challenging, frequently unsuccessful, and potentially leading to unfavorable functional prosthetic defects (4, 6). Unlike the approach of some authors who open the maxillary sinus only in cases of mild to moderate communication (4), radical maxillary sinus surgery was performed in these cases using the Caldwell-Luc technique, due to the presence of extensive inflammatory mucosal changes and sinus empyema. In contrast to Aljohani et al. (4), where sinus was filled with iodophor gauze in cases of combined bone-mucosal defects where the bone defect exceeds 10 mm—requiring an obturator after gauze removal—we opted to completely close the OA communication, although OA communication was extensive, from the tuber and the nasal cavity, using a local mucoperiosteal flap. Furthermore, unlike our case report, Aljohani et al. (4), demonstrated a lower success rate of only 75%, despite having smaller mucosal and bone defects and the presence of communication. The Caldwell-Luc technique is used to improve visualization of the maxillary sinus and remove inflamed mucosa to aid healing and prevent disintegration. In a standard Caldwell-Luc operation, inferior meatal antrostomy is performed to facilitate the postoperative drainage of bloody discharge or sloughs through gravity (7).

In our “FTT Split” protocol the palatal flap was split to a thickness of no more than 3 millimeters to achieve optimal eversion and a 3-millimeter contact between the edges of the palatal and vestibular flaps, and to facilitate easier manipulation of the palatal mucosa. To reduce tension and enable better positioning of the flap, as well as improve contact between the palatal and vestibular mucosa, a suturing technique based on a modified version of Choukroun's horizontal apical mattress sutures is used (22). The key difference in this modification is that the sutures do not end on the vestibular side of the flap, but on the palatal side, with only the periosteum on the vestibular side being sutured. This approach facilitates better flap adaptation, enhances mucosal stability, and reduces flap tension during the postoperative period. Typically, three sutures are placed along half of the palate to ensure even distribution of tension. Vertical mattress sutures help reduce wound tension and minimize the number of sutures required. Additionally, they compress the wound, evert the edges, and provide a larger contact surface for better healing. Everted edges prevent the epithelium from encountering underlying structures, while also tightly adapting tissue flaps to them, such as bone grafts, tissue grafts, alveolar ridges, regenerative membranes, or dental implants (23). Given that the maturation and remodeling phase of mucosal healing begins around three weeks after surgery, and considering potential complications and patient discomfort, the final suture is generally removed three weeks postoperatively (24).

Despite the successful treatment obtained in all patients, this study has several limitations that should be addressed. First, number of patients in this case series is small and only one surgical technique was used, therefore, positive outcomes should be interpreted with caution. Second, although at the follow-ups all patients stated that their quality of life was significantly improved there was no questionnaire that could provide more validated data. Finally, there is a short follow up, with 3 years being the longest period. However given the possibility of disease recurrence, all patients are under further monitoring.

In conclusion, since there are no standardized protocols for the surgical management of MRONJ in maxilla, the “FTT Split” surgical protocol could be considered as one of the surgical treatments for more advanced stages where quality of patient's life is significantly reduced.

## Data Availability

The raw data supporting the conclusions of this article will be made available by the authors, without undue reservation.
